# Community food beliefs during pregnancy in rural kebeles of Ofla Woreda, Northern Ethiopia: an explorative qualitative study

**DOI:** 10.1186/s12884-022-04593-3

**Published:** 2022-03-27

**Authors:** Kebede Eyasu, Lemlem Weledegerima Gebremariam, Freweini Gebrearegay, Zinabu Hadush, Afework Mulugeta

**Affiliations:** Department of Nutrition and Dietetics, School of Public Health, College of Health Sciences, Mekelle University, Tigray, Ethiopia

**Keywords:** Food beliefs, Pregnancy, Qualitative, Tigray, Ethiopia

## Abstract

**Background:**

Dietary related misconceptions during pregnancy affect the heath of mothers and their growing babies. Misconceptions vary from place to place and from community to community. Understanding of a given community’s food perceptions during pregnancy helps policy makers able to design cultural appropriate interventions. In Ethiopia, however, evidences on food beliefs and perceptions during pregnancy are limited. Therefore, this study is aimed at qualitatively assessing community food beliefs during pregnancy in rural kebeles of Ofla Woreda, Northern Ethiopia.

**Methods:**

We conducted 10 in-depth interviews (*n* = 10) and four focus group discussions (*n* = 32) among purposively selected community groups including pregnant mothers, religious leaders, and elders in rural kebeles of Ofla Woreda, Northern Ethiopia. Data were transcribed word-for-word, translated into English, and uploaded into ATLAST ti version 7.5.1.6. Data were analyzed following the principles of thematic analysis. Line-by-line coding was applied to identify codes; identified codes were categorized based on their similarities and differences and themes were developed inductively.

**Results:**

Three main themes were identified inductively; foods positively and negatively linked with pregnancy; perceived benefits and harms of alcoholic drinks during pregnancy; and religion and fasting. In this study, consumptions of animal source foods such as egg were discouraged because such foods were perceived to increase the risk of having big baby that could delay delivery. However, intakes of locally produced alcoholic drinks during pregnancy were encouraged by the local community. Furthermore, avoidance of animal source foods and meal skipping during religious fasting-periods were also common practices among pregnant mothers in the study area.

**Conclusions:**

This study explored misconceptions on food intakes during pregnancy. Positive attitude towards intake of “soft” alcoholic drinks might result in alcohol related teratogenic effects. Restriction on the intakes of nutrient rich diets due to religious fasting and other misconceptions would lead to insufficient nutrient intake both to the mothers and their growing fetus. Culturally appropriate intervention to improve awareness on healthy dietary intake during pregnancy is needed.

**Supplementary Information:**

The online version contains supplementary material available at 10.1186/s12884-022-04593-3.

## Background

During pregnancy, nutritional status of a mother is one of the most important factors associated with adverse pregnancy outcomes [1, 2]. Therefore, women’s energy and nutrients intake need to be improved during pregnancy for optimal growth of the fetus and maternal health [3–5]. The high nutrient and energy demands during pregnancy can be fulfilled through consumption of varied foods and micronutrient supplementations [4, 6, 7].

Inadequate intake of micro—and macro—nutrients during pregnancy leads to negative pregnancy outcomes. Such consequences can also go beyond the immediate negative pregnancy outcomes which can affect the future life of the newborn [8, 9]. As many studies showed that, inadequate intakes of both micro- and- macro nutrients during pregnancy lead to low birth-weight, birth-defects, preterm- delivery, stillbirth, miscarriage, anemia, and maternal death, to mention some [10–13]. Despite the above facts, practice of having low diversified foods and inadequate micro nutrient intakes are commonly seen among pregnant mothers in low- and middle- income countries including Ethiopia. As a result, maternal and child morbidity and mortality is highly prevalent, and avoidable in these countries [8, 14, 15].

In Ethiopia, under nutrition among pregnant mothers is a major public health problem; about 24% of women are anemic [16]. Practice of having diversified food during pregnancy was also found to be low; only about one third, 31.4%, of pregnant women had adequate dietary diversity score, five or more [17], and consumption of folate rich diets was reported only by 8% pregnant women. Moreover, meal skipping and low intake of animal source foods such as meat, fish, and poultry were also reported as common practices during pregnancy [18, 19]. Such poor dietary practices during pregnancy can contribute to the high maternal mortality ratio in Ethiopia. Despite slight decline on the trend of maternal mortality ratio, the country is listed as one of the countries suffering with highest maternal mortality ratio in the world [11, 15].

Maternal dietary practices during pregnancy can be influenced by many factors including lack of knowledge, culture, religion, and societal beliefs and perceptions towards specific foods [20, 21]. A study conducted in four regions of Ethiopia identified that followers of Orthodox and Muslim pregnant mothers observed religious fasts. They also avoided intake of animal source foods and skipped meal throughout the fasting period. Another study also found out that misconceptions related to some foods lead pregnant mothers avoid intake of nutrient rich diets when they are pregnant [19, 22].

Misconceptions and traditions regarding which foods should be avoided during pregnancy and the reasons for avoidance are found to vary from one community to another [23]. Therefore, in order to improve dietary practice during pregnancy in a given community, it is of great importance to understand the local community’s perceptions and beliefs towards dietary habit during pregnancy. Previous studies in Ethiopia, mainly in the study area, however have given more attention on quantifying the magnitude of poor dietary practice during pregnancy [17, 24]. However, community perceptions and beliefs towards intakes of different foods are not well explored. Therefore, the objective of this study was to qualitatively explore perceptions and beliefs towards dietary practice during pregnancy in rural kebeles of Ofla woreda, Tigray region, Northern Ethiopia.

## Methods

### Study setting

This research was conducted in Ofla, a rural woreda, in the southern part of Tigray region, Ethiopia between April and May 2019. The woreda had twenty-one rural kebeles (lowest administrative unit in Ethiopia with a total population of 5,000). Based on the 2007 national census projection, about 144,217 population resided in the woreda of whom 73,550 (51%) were women. Using conversion factor of 3.1% for pregnant women, an estimated 4, 470 pregnant women were residing in the woreda. There were 6 health centers (where curative and preventive health care services were provided) and 22 health posts (lowest health care facilities staffed with two clinical nurses who provided community based prevention services including maternal and child health care services).

Regarding the educational status of the target community, only 9.1% (less than the provincial average that is 15.7%) were literate. Two Kebeles, Hayalo and Zata, were selected purposively for this study after consulting the woreda health office officials. While Zata located far away, about 40 km from a town called Korem, Hayalo is located near by the town. These two kebeles which had different degrees of access to the town that could affect peoples’ perception towards the study subject were selected to explore the different dietary perceptions from different geographic locations and different socio-cultural contexts. The findings help policy makers to understand dietary perceptions from different community groups and design socially and culturally appropriate interventional strategies.

### Study design and participant selection

A community based explorative qualitative study was employed.

Taking into consideration the principles of maximum variation, pregnant mothers who ever gave birth, male and female elders, and religious leaders from Orthodox and Muslim followers were purposively selected for this study. Elders and religious leaders are the ones who guide, advice, and also lead the community. Moreover, they have better understanding about the community beliefs and practices including women’s dietary perception during pregnancy. During participant selection, participants' socio-economic status, educational status, age, and pregnancy status that could enable us to explore their diverse perception towards the purpose of the study were considered. Health extension workers who were working in the target kebeles assisted us in the selection of study participants. Participants were approached on face-to-face base and all contacted participants were willing to participate in the study.

### Data collection methods and materials

Qualitative data for this study were collected from focused group discussions (FGDs) and in- depth interviews (IDIs). A total of ten in-depth interviews (*n* = 10) among pregnant women and four focused group discussions (*n* = 32); one among male elders, one among religious leaders; and two among female elders were conducted. The number of discussants in each group was eight. To facilitate the IDIs and FDGs, topic guides were developed by the authors based on the findings of previously conducted scientific researches (Table S1). Participants’ socio-demographic characteristics were also collected during the data collection period. The guides were developed first in English and then translated into Tigrigna, the participants’ language, and then back translated into English to check for their consistency. The first author, KE, together with ZH who were native speakers of the community’s language and had previous experiences on qualitative research facilitated the IDIs and FGDs using pre-tested topic guides. ZH is an MSc holder academic staff in a University located in the study province and KE is a graduate student in the University. Interviewers provided through explanation on the purpose of the study to the study participants and followed probing techniques to minimize social desirable bias during the interview and discussion. Facilitators were open to accept and consider any opinion that came from the study participants and they did not have predetermined assumptions on the study topic. Before the actual data collection was carried out, topic guides were pre-tested in a different area and comments were incorporated into the final version of the guides. Data collection and analysis were conducted simultaneously. New information from the preceding IDI or FGD were incorporated into the guides and utilized for the next IDI and FGD. Conducting in-depth interview and focus group discussion continued until no new information was found. While all IDIs were facilitated in the participants’ home, FGDs were conducted in Churches, meeting spot, and under a tree where participants could explain their perception freely in natural settings. The average durations of IDIs and FGDs were 45 min and 90 min, respectively. All FGDs and IDIs were audio recorded and field notes were also taken during the data collection period.

### Data analysis

The first author repeatedly listened the audio recordings and read the field notes to familiarize him-self with the data. After familiarization, audio recordings were transcribed word-for-word and then translated into English. Translated data were imported into ATLAST ti version 7.5.1.6, a qualitative data analysis software. After uploading the data into the software, data were analyzed qualitatively following the principles of thematic analysis. First, an initial line-by-line coding was conducted by the first author. Identified codes were discussed among the other authors. Once the identified codes were agreed among the authors, labels were given to each meaningful codes. After that, codes were categorized based on their similarities and differences. Finally, identified categories were grouped to form meaningful themes.

### Validity and rigor

The validity and rigor of the study was assured through different means. First, semi-structured data collection tools were developed by experienced researchers who had diversified areas of expertise including health education and behavioral sciences and public health nutrition both at MSc and PhD level who were academic staffs of Mekelle University. The tools were developed in a way that enable participants speak freely about the subject matter. Second, the importance of maximum variation principles were taken into consideration when participants were purposively selected to make sure findings of the study are coming from different community groups who were considered as potential source of information to the study subject. Furthermore, data collection and analysis were carried out parallel and data collection continued till no new information was generated. Third, data analysis was carried out following the principles of inductive analysis and transcriptions were also done word-to-word (verbatim transcription). Fourth, findings of this stud were triangulate from FGDs and In-depth interviews.

### Ethical consideration

Ethical approval was obtained from the Institutional Review Board (IRB) of Mekelle University. A written informed consent was obtained from each study participant after thoroughly explaining the purpose of the study. Participants were assured that they can leave or stop participating in the study at any time. To ensure confidentiality, all personal identifiers were redacted before data analysis. Participants were assured that the data will be used only for the purpose of this study and will not be used for other study without their approval. All the involvement of humans in this study was carried out following the principles of the Helsinki Declaration.

## Results

### Participants’ socio-demographic characteristics

A total of ten IDIs (*n* = 10) and 4 FGDs (*n* = 32), Each FGD having eight participants, were conducted. While the age of the IDI participants ranged between 21 and 42 years old, the age of the FGD participants ranged between 55 and 70 years old. Almost 62% of the total participants were illiterate, and about 31% attended their primary education and above (Table 1). Only invited participants participated in the study.

### Emergent themes

Following the inductive thematic analysis technique, the following three main themes were identified; foods positively and negatively linked with pregnancy; perceived benefits and harms of alcoholic drinks during pregnancy; and religion and fasting.

### Foods positively and negatively linked with pregnancy

While some foods were perceived as “good” to pregnant mothers, some other foods however were regarded as “not good” to be taken during pregnancy due to the perceive harms both to the pregnant mothers and their growing fetus. To this point, participants of this study identified foods that should and should not be taken during pregnancy and justifications for avoiding some foods during pregnancy.

#### Foods recommended during pregnancy

Participants of this study emphasized the importance of having balanced food during pregnancy. They believed that having balanced food help pregnant mothers get energy and properly nourish their unborn baby. Most IDIs and FGD discussants explained that liquid foods such as milk, soft drinks, “muk’’ or “atmit” (soup), “telba” (linseed), porridge, slightly warmed honey, water and cereals such as rice, teff, “kik” (grits of legumes like bean) are foods which are important for pregnant women as they help pregnant woman to get adequate energy and get hydrated. Such foods were also reported as helpful for “safe” delivery, facilitate delivery, prevent pregnant women’s body from “dryness”, act as lubricant during delivery, shorten labor time, and prevent “mit” or “chirmi” (labor pain).

Unlike roasted chickpea and wheat which were regarded as “bad foods”, roasted barely was reported as one of the energy providing foods that causes minimal or no spasm and it can be crashed easily by human teeth. Moreover, fruits and vegetables such as cabbage, lettuce, banana, carrots, and potatoes were also perceived as “good” foods for pregnant women. They reported that the above mentioned foods are mainly important during the last trimester of pregnancy as they can help pregnant mothers get energy so as to push the baby out during delivery. They also believed that when delivery time is approaching, pregnant mothers should take easily digestible foods and liquid foods.

“*For me, it is important to prevent pain during delivery and shorten labor duration. If I don’t drink liquids, it will endanger me. Energy providing meals will be helpful for me during delivery. Milk gives energy and liquids prevent the body from dryness. If you don’t drink water […..] your body gets dry. […] liquids like milk and soft drinks prevent our body from dryness. They lubricate and soften my body so that labor pain and time during delivery will be minimal or it will be easy.”*

*IDI, 28 years old pregnant woman*

*“After four months of pregnancy, the child starts to grow. Hence, the mother has to eat balanced diet. When the delivery time is approaching, pregnant women should take easily digestible foods and liquids.”*

*FGD, 69 years old man*

#### Foods not recommended during pregnancy

Participants of this study also identified foods that are not recommended during pregnancy. Foods such as egg, “kolo” (roasted wheat or chickpea), red and green pepper, hot drinks like hot milk, and mustard containing foods, were identified as foods that should be avoided during pregnancy. Intakes of such foods were perceived to affect the health of both the mother and the unborn fetus. Elders in the FGDs reported that egg is believed to produce belching and fattening of mother and it sticks on the face of the baby results for the delivery of “unclean” baby. Eating “kollo” (roasted wheat or chickpea) was also believed to cause abdominal spasm to the child and the mother, makes labor painful and agony, and the child will be born with a crying baby. All participants also believed that mustard containing foods are absolutely forbidden for pregnant women as such foods were perceived to terminate the pregnancy. It was believed that mustard aborts the child immediately when it is eaten by pregnant mother. Some respondents also believed that having paper during pregnancy is harmful as it was believed to “burn” the fetus and narrows the eyes of the fetus.

*“Mustard is poison and you should avoid eating a given food if you know it contains mustard. It can injure the baby and even the mother. It is strong poison. I can say it is acidic. If I use these foods, my baby will get out. There are women who miscarriage their baby by drinking “senafich” (mustard). Pepper makes child’s eye “chemcham” (narrow eye). But its acidity is not like mustard.”*

*IDI, 25 years old pregnant woman*

*If she eats dry foods like “kollo”, eggs, sugarcane and “muk”, they will not digest and directly go to the child. These foods may attach to the baby’s body which leads to have unclean baby and painful and long labor.*

*FGD, 64 years old woman*

*“Chickpea and wheat kollo are not good for pregnant women. They cause cramping and spasm. They also increase duration and pain during labor as they are in their original form. They are not milled. If she (pregnant mother) needs to eat “kolo” it must be barley kollo. It is energy providing since it is soft and it can be crashed and milled by the teeth of pregnant women.”*

*FGD, 42 years old woman*

### Perceived benefits and harms of alcoholic drinks during pregnancy

Participants of this study have classified alcoholic drinks into “dry” and “soft”. While intake of “soft” alcoholic drinks during pregnancy were perceived as beneficial, intake of “dry” alcoholic drinks were regarded as harmful.

#### Perceived benefits of “soft” alcoholic drinks

Participants reported that alcoholic drinks with less alcoholic content are named as “soft” and they perceived that intake of such alcoholic drinks during pregnancy are important both to pregnant mothers and their growing fetus. They perceived that “tella” and “korefie” are beneficial to “clean” the fetus, “clean” the uterus before and during pregnancy, and “hydrate” the body of pregnant women. Drinking “tella” was also believed help to have “a neat child” as it is believed that “tella” would “clean” the fetus while he/she is in his/her mother’s womb. As a result, informants reported that pregnant mothers are recommended to take such alcoholic drinks.

#### Perceived harms of “dry” alcoholic drinks

Participants of the IDI and FGD perceived that alcoholic drinks with high alcoholic contents such as Arekie” and “katikala” are named as “dry” alcoholic drinks. Almost all participants agreed that such alcoholic drinks should be avoided by pregnant mothers especially during late trimester of pregnancy as they were perceived to harm both the child and the mother.

“*Dry alcohols like “arekie” are prohibited since 40 days of pregnancy.”*

*FGD, 42 years old woman*

### Religion and fasting

Observing religious fasting was a must even among pregnant women in the study area. It was considered as a sin and nonreligious act to break fasting by most of the participants. All pregnant women informants believed that fasting is a guarantee for them to safely deliver and avoid problems during delivery that could happen due breaking religious rules including fast. As a result, they avoid milk, eggs, meat, and butter containing foods throughout the fasting period. Moreover, most of them didn’t take breakfast; they fast at least up to six o’clock local time (12 pm), without taking any foods including water.

*“I go every morning to church and I learned that fasting is pillar to me during pregnancy. It is guarantee for pregnant women in order to deliver safely. It is also a fence which protects pregnant women from different problems. If you are Christian, you have to fast. It is clear that the holy book doesn’t allow us to take milk or meat even you are pregnant. If you break fasting, it is a must to confess to your God father in order to atone.”*

*IDI, 35 years old pregnant woman*

Elders and Orthodox Christian religious leaders explained that fasting is a religious act and they cannot discourage pregnant mothers against fasting. They reported that breaking fasting is allowed only to mothers who have given birth (after delivery) and during the first months of nursing. Breaking fasting can be allowed to pregnant women only when it is recommended by health care providers due to illness, medication or other special requirements. Religious leaders illustrated that they can allow pregnant women to have their breakfast. However, it was very difficult for them to allow pregnant women to have milk and other animal source foods, meat and eggs during fasting periods. They believed that having such foods during fasting periods is out of the Gospel. They revealed that teaching against fasting is also out of their religious Gospel. They also addressed that even if they allowed pregnant women to break fasting, the women would refuse to take meat, egg and milk during fasting periods.

*“During fasting, we don’t encourage (if we don’t prohibit) pregnant women to take foods containing milk, meat, egg and butter. We can’t go out of Gospel to allow pregnant women to take such foods during fasting periods. Even the women themselves think the life and soul of pregnant women is on the hands of God and fasting is the guarantee to deliver safely.”*

*FGD, 67 years old man*

Religious leaders of Muslim followers and elders explained that it is possible for a pregnant woman to postpone fasting. Mothers could compensate it after they gave birth. They also perceived that if pregnant women are healthy, they are expected to fast. The elders reported that, Muslim followers do not have any food to avoid during religious fasting period. However, fasting women are expected to abstain from food and fluid including water from 6 am to 7 pm.

## Discussion

This exploratory qualitative study identified food beliefs that influence dietary practice of pregnant women in rural districts of Ofla, Tigray province, Ethiopia for the first time.

The study revealed that energy dense foods and pulse foods which were described as ‘lubricating foods’, such as linseed, soft drinks, porridge, honey, cereals, and vegetables and fruits were regarded as good foods as they were perceived to speed up delivery, help to have safe delivery, and minimize labor pain through ‘softening’ the uterus and birth canal. They were also believed to improve maternal physical strength during pregnancy so that mothers could have ‘enough’ energy to push out the baby and to have a healthy baby. Such perceptions were similar with findings of previously conducted qualitative studies in Ethiopia [19, 25–29] and rural Kenya [30, 31]. However, in some studies consumption of linseed by pregnant mothers was restricted [25, 27] and in some others milk was restricted [25]. This might be due to differences in culturally constructed perceived reasons of recommending or restricting specific foods during pregnancy [32].

It is obviously known that dietary related misconceptions modify peoples’ dietary intake and affect their lifestyle [25]. Regarding dietary related misconceptions during pregnancy, their effects go beyond the maternal health and could affect the outcomes of the pregnancy and they determine the future life of the fetus [33]. In our study, on one hand, intake of animal source foods such as meat, milk, and butter were reserved for postnatal periods. On the other hand, pregnant mothers were prohibited to have some food types such as “kollo” (roasted wheat or chickpea), animal source foods such as egg; green pepper; and mustard containing foods throughout their pregnancy period as these foods were believed to cause abdominal cramp both to the baby and mother, and dehydration which can result in delayed delivery (“kollo” (roasted wheat or chickpea). They were also perceived to cause fattening for the mother result in having a big baby (egg). Such misconceptions were reported in findings of previously conducted studies [20, 32]. Avoidance or minimal intake of whole grains such as “kollo” (roasted wheat or legumes) affect fiber intake of pregnant mothers which could increase the risk of developing gestational diabetes mellitus [34]. Furthermore, avoidance of animal source foods such as egg by pregnant mothers could lead to poor weight gain during pregnancy, low birthweight, embryonic losses, intra-uterine growth restriction, and reduced postnatal growth due to a deficiency in specific amino acids that are important for cell metabolism and function [26, 35, 36].

Although evidences on the health benefits and risks of alcohol intake among adults are debating [37], the negative health impacts of alcohol intake during pregnancy both to the unborn fetus and the mother are clearly stated in many studies [38, 39]. In this study, however, participants’ perception towards alcohol intake during pregnancy was determined based on their perceived classification of alcohols. To this point, while some alcohol types which were perceived as “soft alcohols” such as beer and “tella” were recommended to be taken during pregnancy, others which were regarded as “dry” alcohols to mean that strong alcohols such as ‘arekie’ and ‘katicala’ were restricted during pregnancy. Beer and “tella” intake during pregnancy was recommended in a study conducted in Bahir-Dar [40]. The teratogenic effect of alcohol consumption during pregnancy have been reported in a scientific studies and its effects have proved to cause low birthweight and small for gestational age [41].

Despite the endorsement of Nutrition Sermon Guide by the Patriarch of Ethiopian Orthodox Tewahdo Church to encourage nutritious food intake including animal source foods for pregnant mothers and children during fasting period is there [42], pregnant mothers in the study area however, have reported that fasting is practicing among them as part of their religious obligation. As a result, animal source foods intake during pregnancy was prohibited in addition to skipping meal during fasting period. Muslim observer pregnant mothers also reported that any kind of food and fluid intakes are prohibited during fasting, Ramadan, period from 5am to 7 pm. Such fasting practices among pregnant mothers have been reported in previous studies [21, 22]. While some studies reported the positive health benefits of religious fast among adults [43, 44], its effect during pregnancy is not fully explored [45].

### Strength and limitation of the study

This is the first study that explored food beliefs during pregnancy from different community members’ perspective. Findings were triangulated from IDIs and FGDs. However, this study had some limitations; the first limitation was that, although the IDIs and FGDs were carefully conducted, the effect of social desirability biases were unavoidable Second, preliminary findings of the study did not sent out to the participants of this study for members’ check.

## Conclusion

This study explored misconceptions on food intakes during pregnancy in the study area which can negatively harm the health of pregnant mothers and their growing fetus. The positive attitude towards intake of “soft” alcoholic drinks would result for the unnecessary alcohol intake during pregnancy. Such alcoholic drinks during pregnancy might result into different teratogenic effects including intrauterine growth restrictions and low birthweight. Furthermore, restrictions toward the intakes of nutrient rich diets due to religious fasting and other misconceptions would also lead to insufficient nutrient intake both to the mothers and their growing fetus. Inadequate nutrient intakes might result into both short and long-term undernutrition related complications. Therefore, it is need to design culturally appropriate health education and promotion to improve awareness on healthy dietary intake during pregnancy.

**Table1:** Characteristics of the IDI and FGD participants

Socio-demographic Characteristics	IDI (*N* = 10)	FGD (*N* = 32)
**Age**		
20–29	7	0
30–44	3	6
45–49	0	6
≥ 50	0	20
**Sex**		
Male	0	16
Female	10	16
**Educational status**		
Illiterate	3	26
Primary Education	4	4
Secondary Education	3	2
**Marital status**		
Married	9	29
Single/divorced	1	3

## Supplementary Information

**Additional file 1.** FGD and IDI topic guides

**Additional file 2.** COREQ checklist-Food beliefs

## Data Availability

Data, translated data from the audio records and field notes, which were analyzed during this study, will be available from the corresponding author upon reasonable request.

## Abbreviations

FGD: Focus group discussion
IDI: In-depth interview
