# Supplementary material for: Community food beliefs during pregnancy in rural kebeles of Ofla Woreda, Northern Ethiopia: an explorative qualitative study
Source: BMC Pregnancy Childbirth. 2022 Mar 27;22:256. doi: 10.1186/s12884-022-04593-3 (PMC8958790; doi:10.1186/s12884-022-04593-3)
Supplement: Supplementary file 1 — Additional file 1. FGD and IDI topic guides [file 12884_2022_4593_MOESM1_ESM.docx]

# Table S1: In-depth interview and focus group discussion topic guides

**In-depth interview topic guide**

Introductory question: - How to you feel your dietary practice while you are pregnant?

Main points: - Which foods should be eaten during pregnancy?

Probe: could you elaborate it how do you think such foods benefit pregnant mothers including yourself?

- In your opinion, which foods should be avoided during pregnancy? Probe: could you elaborate it why do you think they should be avoided by pregnant mothers?
- How do you think such foods affect pregnant mothers including yourself?
- How do see your dietary practice in relation to your opinion? Could you practice in a way how perceive it should be?

Probe: Could you elaborate on how you practice it/not practice it and why?

- How do you/other pregnant mothers practice your/their diet during religious fast?

Probe: Any food restriction, meal frequency

**Focus group discussion topic guide**

- What do you know about pregnant women’s dietary practice in your community?
- How do pregnant mothers’ dietary practices look like?

Probe: which foods/drinks are commonly eaten during pregnancy? How such foods could help the mother? Would you give me an example please?

Probe: any foods/drinks that restricted during pregnancy? How do you think such foods affect the mother? Would you give me an example please?

- How should be the food practices of women during Pregnancy? Why it should be like that?
- How should pregnant women eat during religious fasting periods?

Probe: Food Types and meal frequency
